# The feasibility and acceptability of a preventive intervention programme for children with depressed parents: study protocol for a randomised controlled trial

**DOI:** 10.1186/s13063-016-1348-7

**Published:** 2016-05-06

**Authors:** Valeria de Angel, Fernanda Prieto, Tracy R. G. Gladstone, William R. Beardslee, Matías Irarrázaval

**Affiliations:** Department of Psychiatry, Hospital Clínico University of Chile Medical School, Avenida La Paz, Recoleta, Santiago 1003 Chile; Wellesley College, 106 Central Street, Wellesley, MA 02481-8203 USA; Department of Psychiatry, Baer Prevention Initiatives, Boston Children’s Hospital, 300 Longwood Avenue, Boston, MA 02115 USA; Millennium Institute for Research in Depression and Personality, Avda. Vicuña Mackenna, Macul, Santiago 1003 Chile

**Keywords:** Prevention, Depressive disorder, Parental depression, Family-based, Children, Adaptation, Feasibility, Acceptability, Protocol, Pilot

## Abstract

**Background:**

One of the most important risk factors for childhood depression is being the child of a depressed parent. These at-risk children have two to four times the probability of having an affective episode compared with their peers. Preventive interventions such as Beardslee’s Preventive Intervention Program (PIP) that are targeted at children of depressed parents have proven effective in many countries. The PIP is a family-based approach that works by promoting resilience in children and increasing positive interactions within the family. In this pilot randomised controlled trial (RCT), we will determine the acceptability and feasibility of an adapted version of this intervention in Chile.

**Methods/design:**

We are conducting a pilot RCT with a manualized intervention. The intervention will be delivered in seven weekly sessions at the family home. It is targeted mostly at parents but will also measure outcomes among the children. Control subjects will follow their treatment as usual. Feasibility and acceptability will be assessed by recruitment, adherence, dropout and level of missing data, as well as the burden of scales and measurement tools. Families will be followed for 11 months.

**Discussion:**

Given the negative lifelong consequences of depression and the burden they represent, preventive programmes are not only feasible but necessary. Despite the successful implementation of this intervention in different countries, an adaptation to the Chilean reality will be prerequisite. The results of this pilot study will inform a definitive trial that will make the case for its national implementation.

**Trial registration:**

Clinicaltrials.gov trial identifier: NCT02593266. Registration date: 30 Octo 2015.

## Background

Major depression is a prevalent and disabling condition that constitutes an important public health problem in Chile. This growing problem has been identified by numerous organizations as a major health-care priority in the country [1].

In the Chilean population, depressive disorders represent the second leading cause of specific disease [2]. Its prevalence, according to the latest national health survey, is 17.2 %, and is higher in women with fewer than 8 years of education [3]. More than 30 % of women consulting in primary health care have a diagnosis of depression. It is the second leading cause of disability in Chilean women, as measured by disability-adjusted life-years, and the fourth leading contributor to the global burden of disease [4, 5]. This disorder leads to greater disability than other chronic diseases, such as hypertension and diabetes, and explains over 40 % of sick leaves [1].

Depression is not a problem that affects only adults. In fact, 8 % of adolescents in Chile have major depression at any given time, and the lifetime prevalence of depression is 14 % [6]. Using the Beck Depression Inventory-II (BDI-II), researchers in a study in the city of Concepción found that 32.6 % of 746 secondary school students had depressive syndrome [7].

One of the most important risk factors for childhood depression is being the child of a depressed parent [6]. In fact, by age 20, a child with an affectively ill parent has a 40 % chance of having an episode of depression, and by age 25, this rate increases to 60 % [8]. These rates are two to four times higher than rates among their counterparts from homes without parental illness [9, 10]. Having a parent with depression is also associated with an increased risk of anxiety disorders and a variety of other problems, including poor health and deficits in academic performance, social relationships and self-esteem [10, 11].

One possible environmental mechanism for the transmission of depression from parent to child is the quality of the parenting that the child receives [12, 13]. Parental depression negatively affects caregiving, material support and nurturance. Observational studies have documented numerous parenting difficulties among depressed mothers, including increased hostility, higher rates of negative interactions and impatient use of directives in guiding their children’s behaviour [13–15]. In other studies, depressed mothers have been found to be less responsive to their children’s behaviour, to communicate less effectively, to demonstrate lower synchrony with their infants, and to have fewer positive interactions with their children [16, 17]. In turn, quality of parenting has been found to be related to depression in children and adolescents (see [18] for a meta-analysis).

All of this, coupled with the fact that there is a big gap between the population of adolescents in Chile who manifest some form of mental health disorder and the population of adolescents who ultimately receive treatment, creates a very large need for preventive strategies.

*Prevention* refers to interventions before the onset of a disorder that are designed to prevent the disorder’s occurrence [19]. Preventive strategies are aimed at reducing the incidence, prevalence and recurrence of mental disorders; the time spent with symptoms; the risks for such mental illnesses; and the effects of illness on affected people, their families and society [20]. Recent efforts to develop preventive interventions for depression have been successful and suggest that depression is amenable to a public health approach to disease prevention [21, 22]. A preventive approach to mental illness is not only desirable but feasible and, according to a recent meta-analysis, also cost-effective [23].

To date, mental health interventions for depression have been focused almost exclusively on an individual treatment approach, but selective preventive interventions that are targeted at community individuals or groups demonstrating a higher than average risk have a strong evidence base [24]. A recent meta-analysis by Siegenthaler and colleagues of randomised controlled prevention trials showed that, in children of parents with a mental disorder, the risk to offspring for the same mental disorder was reduced by 40 % [25].

There are a number of selective prevention strategies designed specifically for children of parents with depression that have been successful, and that have in common a focus on strengthening parenting and addressing youth needs. Beardslee and colleagues reviewed important national programmes such as those of Finland and Australia [26].

In Finland, the Effective Child and Family Programme is supported by the Ministry of Social Affairs and Health, which intends to make a system change in health and social services so that professionals can attend to the needs of adult patients and their children. The programme incorporates families with psychiatric problems to promote children’s well-being and to prevent children’s problems. Some of the strengths of the programme are its multilevel structure and the application of family intervention sessions similar to those of the Beardslee Preventive Intervention Program (PIP) for Depression [26].

Australia has developed the Children of Parents with a Mental Illness national initiative to support families with a parent with depression. Here, a version of the PIP for Depression was used with the Family Talk technique. Systematic approaches, such as those used in Finland and Australia, offer the best opportunity for large-scale impact, and there are important strategies that practitioners can employ directly with families [26].

### The Beardslee Preventive Intervention Program for Depression

Beardslee’s PIP is one of few methods in the field that uses family-based methods developed for preventive practice among children of parents with depressive disorders and mental illness [27]. It has been implemented in Sweden and Finland on a national level, and it also has been successful in the Netherlands, Norway, Sweden, Colombia, Costa Rica, Iceland, and Chicago in the United States [28].

The main purpose of the PIP is to prevent depression and other mental health problems in children of depressed parents by promoting resilience of children and increasing positive interactions within the family. Resilience is the reason why many children of depressed parents do not develop symptomatology; it enables them to accomplish age-appropriate developmental tasks, engage in relationships and understand their surroundings despite their parents’ illness [29, 30]. This programme generates opportunities for resilience promotion in children by supporting families to identify and build upon these resilience processes. Resilience is an important protective factor that can counterbalance the multiple risk factors for depression among children and adolescents, such as by developing the ability to understand their parents’ illness and move on [31]. On the other hand, the parents’ resilience that this programme builds upon is the capacity to remain committed to parenting despite depression and to reflect on how they could become effective parents [32].

The PIP’s aim is to provide psychoeducational material, to link that to the family’s life experiences; to increase understanding within the family; and, in that way, to help them find their own strengths, begin to plan to overcome depression and be able to have family conversations about depression. Negative interactions are one of the proximal mechanisms through which the effects of parental depression are transmitted to children [33]. Including conversation skills in this intervention, improving communication about parental illness in the family, promoting other protective factors for the children, and strengthening the parents in their role as caregivers are strategies also used for enhancing resilience [34]. Communication and openness about the parent’s illness within the family improves children’s understanding of parental illness and their own situation—also a characteristic of resilient adolescents [30]—and enhances parental and family functioning. Parents are also provided with information about the other well-known protective factors for the children, such as school, friends and interests, and are encouraged to support these.

The PIP is a manualised method available in Spanish that has been offered in two public health formats: two lectures for parents and a seven-session intervention called Family Talk. In the latter version, both parents are given an opportunity to talk about their experiences and the impact of the illness on themselves and their children. Parents’ concerns about their children are paid attention to, as are their questions about the children. Each child is then interviewed individually with a focus on the child’s experiences of the parent’s illness. Protective risk factors for the child are assessed. Subsequently, parents are given feedback from the child’s interview, and a family session is planned with the children’s questions and experiences as a basis. In the family session, parents are encouraged to talk about their illness with the children by themselves in order to start a process towards mutual understanding within the family. Follow-up after 1 and 6 months is recommended, with the aim of checking on the family, addressing concerns and reviewing protective factors for the children.

Beardslee’s family intervention has evidence of long-term effects for children and their families [35–37]. Outcomes included diminished adolescent internalizing symptoms, increased positive family interactions, and increased rates of recognition and treatment for children who did eventually experience depression [38].

Given the prevalence and burden of depression in Chile, a preventive intervention such as the PIP for Depression is much needed. This intervention needs to be adapted and integrated into the Chilean reality first.

### Aims and objectives

The aim of this study is to evaluate the acceptability of an adaptation of Beardslee’s PIP for Depression in Chilean families. Additionally, we will test the feasibility of implementing this programme broadly. The main objectives therefore are as follows:

- To determine whether the PIP for Depression is acceptable for Chilean families
- To assess recruitment processes and study uptake to inform the feasibility of a full-scale randomised controlled trial (RCT)
- To evaluate the burden of scales and measurement tools
- To measure response, dropout rates and level of missing data

The effects of the family intervention on parental depression will be evaluated. Internalising and depressive symptoms in children and their global psychosocial functioning will also be measured.

## Methods/design

### Setting

The first evaluation will happen at the families’ nearest mental health centre; subsequently, the intervention will be carried out in people’s homes. Families will all be living in Recoleta, a 16-km^2^ urban subdivision of Chile’s capital city of Santiago comprising 148,220 habitants. Within this catchment area, participants will be contacted via the five mental health centres available.

### Design

The present study is a pilot trial and will therefore be primarily exploratory. It will provide qualitative information in terms of how feasible and accepted the PIP for Depression could be for Chilean families. The design of the quantitative side of the study will be a single-blind RCT. The intervention group will receive PIP for Depression at their home in addition to their usual treatment for depression, whereas participants in the control group will be on a waiting list for the intervention and will receive their treatment as usual (pharmacotherapy and/or psychotherapy).

### Primary outcomes

Acceptability will be assessed by determining how well the intervention is received by the target population and the extent to which this new intervention might meet the needs of the target population. Feasibility will be ascertained by looking at whether the aims of the study are achieved and appraising whether, judging by the general experience in the pilot study, an upscaled project is likely to succeed. The recruitment and intervention processes will be scrutinised.

### Secondary outcomes

The following are secondary outcomes to be considered:

1. Depressive symptoms in both the child (Children’s Depression Inventory) [39] and the parent (BDI-II) [40]
2. Family function (Family Adaptability and Cohesion Evaluation Scales [FACES]) [41]
3. Parental competence (Positive Parenting Scale-Second Edition [E2P]) [42]
4. Adaptive behaviour in the child (Child Behaviour Checklist [CBCL]) [43]
5. Resilience in the child (School Resilience Scale [ERE]) [44]
6. Cognitive function (Wechsler Intelligence Scale for Children-Third Edition [WISC-III] Processing Speed Index, which includes coding and symbol search subtests) [45]

The hypotheses for the intervention are as follows:

1. Children in the intervention group will not show an increase in depressive and internalising symptoms as compared with those in the waiting-list control group.
2. Parents in the intervention group will have increased their family functioning scores and decreased their general psychopathology scores compared with those in the control group.

### Materials

#### Sociodemographic questionnaire

A brief questionnaire was designed to collect sociodemographic information on the parents and their children. Questions on age, gender, education level, occupation and work status of every person living in the household were included. Information on household income in the last year and the last month was also obtained.

#### Qualitative measures

Acceptability will be measured via qualitative feedback forms after each session. These forms will include questions on the participants’ thoughts and opinions about the intervention content and its delivery. They will include items such as, “How relevant to you and your family’s issues was this module?”, “How satisfied were you with today’s session?”, and “In your opinion, is there anything that prevented the session from suitably taking place?” The feedback obtained from this questionnaire will be used to continuously optimise the intervention. Furthermore, at the end of the intervention, focus groups will be created for families and therapists. Forms designed to determine which components of the intervention are covered in each session will be completed to ascertain the integrity of the intervention and adherence to the manual. To further examine the acceptability of the intervention and adherence to the manual, 20 % of the total number of sessions in the studies will be videotaped for those families who give their consent.

The feasibility of this pilot study requires that the intervention be effectively carried out with an adequate number of families and within the delineated time. The feasibility of the recruitment process will include the number of families sampled and contacted, those families who accept, the number of families who complete the intervention and reasons for ineligibility, and non-participation or early withdrawal from intervention. The feasibility of the intervention will include the number of sessions attended, time taken to complete questionnaires (burden of questionnaire completion), items left blank on questionnaires, follow-up rates and dropout. The number of therapists available and how many families they manage to visit per week will also be noted. As it is important to evaluate whether the administration and assignation of resources is effective, supervisors at health centres will also be interviewed.

#### Quantitative measures

The following quantitative measures will be used:

- M.I.N.I.: The Mini International Neuropsychiatric Interview (M.I.N.I.) version 5.0 [46] is a brief structured diagnostic interview developed in 1990 by doctors and psychiatrists in the United States and Europe. With a delivery time of approximately 15 minutes, the M.I.N.I. 5.0 is a diagnostic tool for psychiatric disorders based on the *Diagnostic and Statistical Manual of Mental Disorders, Fourth Edition* (DSM-IV), and the International Classification of Diseases, Tenth Revision.
- BDI-II: The BDI-II is a self-assessed multiple-choice scale consisting of 21 questions. It is one of the most widely used instruments in the world that measures the severity of depression. It is designed for people older than 13 years of age and consists of items that measure depressive symptoms, hopelessness, irritability, guilt and physical symptoms.
- E2P: The Positive Parenting Scale (E2P) is a self-administered 54-item questionnaire on parental skills which covers four subcategories: bond, development, protection and self-reflection.
- FACES-II: This is the second version of FACES, a self-administered scale measuring cohesion and adaptability in the family. There are 50 items, 25 of which are intended to explore the dimension of cohesion and the remaining 25 adaptability.
- MINI-Kid [47]: The Mini International Neuropsychiatric Interview for Children and Adolescents (MINI-Kid) is the children’s version of the M.I.N.I. The structure is identical, but the questions are modified to facilitate the understanding of children.
- CDI: The Children’s Depression Inventory assesses depressive symptoms and provides a total score for depression in children. It may be self-administered by the children, but in this investigation it will be administered by an evaluator. It consists of 27 items, each consisting of 3 statements, and measures 2 dimensions: dysphoria (depressed mood, sadness, worry, and so forth) and negative self-esteem (judgments of inefficiency, ugliness, evil, and so forth).
- CBCL: The Child Behaviour Checklist scale is completed by parents to detect emotional and behaviour problems in children and adolescents. It consists of 113 questions, scored on a 3-point Likert scale (0 = absent, 1 = happens sometimes, 2 = often occurs). This scale provides information on the following syndromes: anxiety, depression, somatic complaints, social problems, thought problems, attention problems, and rebellious and aggressive behaviour. Also, it has six scales based on DSM-IV diagnostic categories: emotional problems, anxiety, somatic problems, attention-deficit/hyperactivity disorder, oppositional defiant problems and behaviour problems.
- School Resilience Scale (ERE): This scale contains 27 items for children between the ages of 9 and 14 years. The ERE measures the level of overall resilience of children and pre-adolescents in three main areas (I am, I can and I have) subdivided into five dimensions: self-identity, network models, learning generativity, internal resources and external resources.
- WISC-III: Two subscales of the WISC-III will be administered to measure processing speed. In the coding test, there is a series of simple forms (coding A) or numbers (coding B), each of which has a corresponding code, and the child is asked to draw the symbol under its corresponding form or number according to a key. In the symbol search test, the child is asked to decide whether target symbols appear in a row of symbols.

### Participants

The sample will be obtained from among all patients with depression who have attended and are registered in the five mental health centres in the Recoleta area.

#### Inclusion criteria for families

Patients will be eligible if they are currently undergoing a depressive episode or if they have done so in the past 3 months. Eligible patients must have at least one non-depressed child between the ages of 6 and 12 years. In families in which only one parent has a depressive disorder, this parent will be the “identified parent” (IP) and the parent who does not have a disorder will be the “unidentified parent”. If both parents have depression, the parent with the most severe or chronic mood disorder will be designated as the IP.

#### Exclusion criteria

The following are the exclusion criteria for parents:

1. Alcohol or drug dependence or abuse as assessed with the M.I.N.I., or being in treatment for substance use
2. Psychosis, personality disorder, bipolar disorder or suicide ideation as assessed with the M.I.N.I.
3. Having a relationship crisis with a current partner, assessed by self-report or by having attended couple’s therapy in the past month
4. Attending family therapy

The following are the exclusion criteria for children:

1. Being outside the age range of 6–12 years at the time of recruitment
2. Inability to understand or answer age-appropriate questionnaires (due to intellectual disability or otherwise)
3. Having depression as assessed with the M.I.N.I.-Kid
4. Being in treatment for or having taken psychotropic medication in the last month

#### Therapist recruitment

The people recruited to carry out the intervention will be psychologists and social workers in the different health centres as well as in schools within this catchment area. They will undergo a total of 9 h of training with a final assessment as an indicator of their abilities. It is expected that 20–25 people will be successfully trained in the intervention.

#### Sample size calculation

Despite the fact that a power calculation is not fully appropriate for a feasibility study [48], we have estimated a suitable number of children for this study. We expect that the proportion of children who will develop depressive symptoms within 1 year in the waiting-list control group will be 0.33, compared with 0.05 for those children in the intervention arm. For a loss to follow-up calculated at 15 % and in a two-sided contrast analysis, 32 children will be required per arm and 64 children in total. Despite there being evidence to support a one-sided analysis for this case, since we assume the intervention will not cause harm, we will carry out a two-sided analysis as is common in RCTs. This number is adequate according to recommendations for feasibility studies which propose a minimum of 30 participants per arm to estimate parameters for future sample size calculations [49, 50].

### Procedures

#### Recruitment

**Fig. 1:**
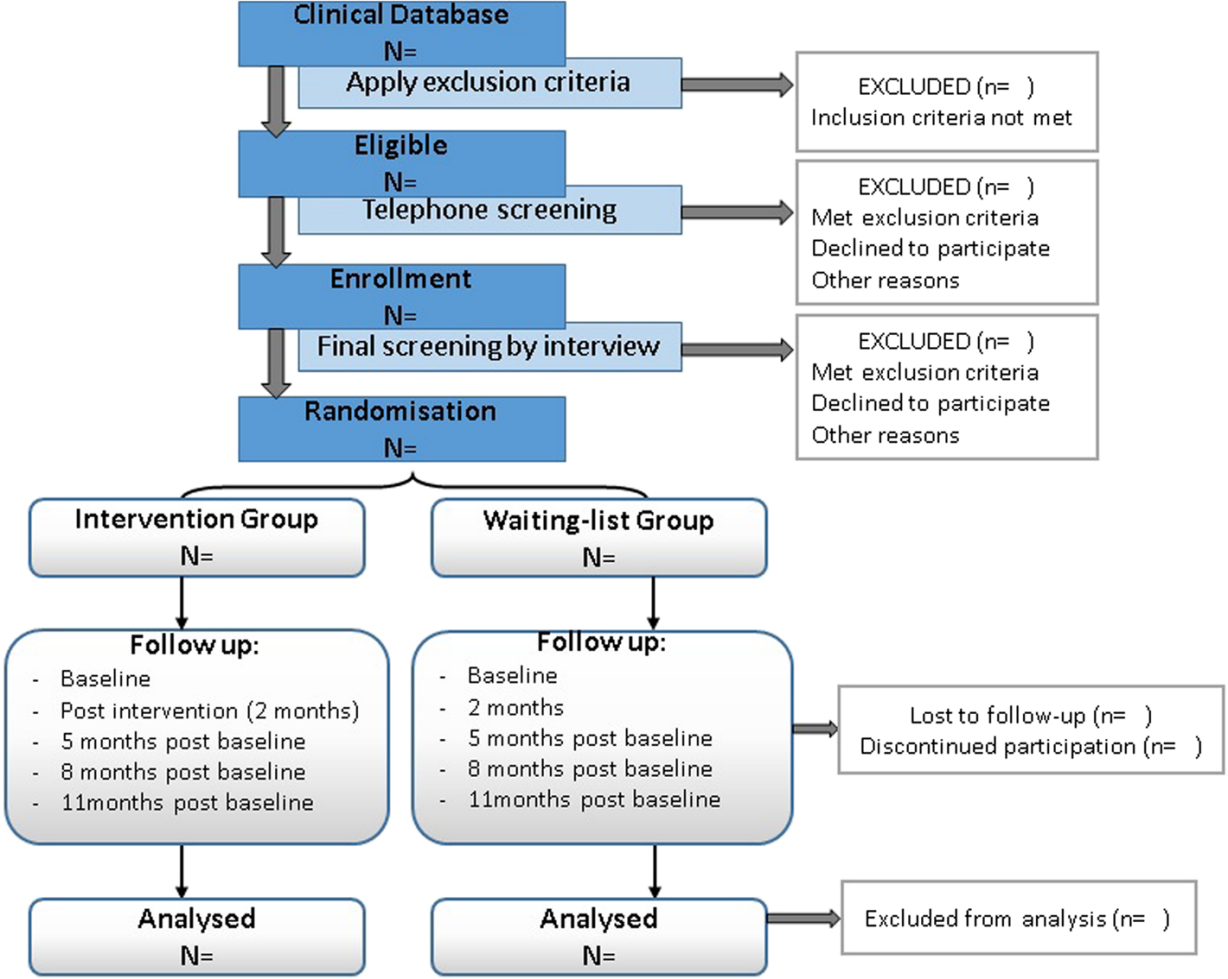
Flowchart of study design and participant flow, from enrolment to final follow-up

The flowchart displayed in Fig. 1 summarizes the recruitment procedure. Patients attending health clinics for depression in the catchment area will be contacted by telephone and invited to participate in the study. Those who accept will be asked to answer a few screening questions on the telephone and, if they meet the inclusion criteria, will be invited for the primary assessment at their usual health clinics. This first assessment will involve further information on the study, clarification of any doubts or questions that prospective participants may have, and signing of the informed consent forms. After this, final screening measures that can only be done face-to-face will be administered. If eligible, participants will be assigned alphanumeric participant codes, and baseline evaluations will be conducted.

#### Randomisation

Once the family is fully enrolled and baseline evaluations are completed, the family will be randomised to either the intervention arm or a waiting-list group by a collaborator who will be blind to all evaluation outcomes and will conceal the allocation sequence until the intervention is assigned. The randomisation will be done with the use of a random number generator and will be stratified by health centre catchment areas. The use of block randomisation means that the intervention can be delivered in a staggered manner, with some participants beginning the intervention while further recruitment continues.

#### Blinding

The primary outcomes involve intervention-specific questions, so blinding becomes impossible. For the secondary outcomes, however, evaluators will be blind to the treatment allocation as well as those carrying out the statistical analyses. The randomisation and statistical analysis will be carried out by someone unconnected to the recruitment process.

#### Intervention arm: Family Talk PIP for Depression

A manualized intervention will be carried out, designed by Beardslee and his team. This is a preventive intervention programme for depression aimed at families in which one or both parents have depression. It is family-centred and focused on the strengths of the family. Based on cognitive, narrative and systemic therapy, Family Talk aims to strengthen family relationships, functioning and communication. It is divided into seven modules that will be delivered weekly (plus follow-up):

- Module 1: Depression and family
- Module 2: Psychoeducation about depression
- Module 3: The perspectives of children on their parents’ depression
- Module 4: Skills development
- Module 5: Preparing for family meeting
- Module 6: Family meeting
- Module 7: Review and future planning
- Follow-up

Each module is a 60- to 90-minute meeting wherein parents learn about depression, discuss their experiences and develop coping skills.

#### Waiting-list control group

Families assigned to the control group will carry on with their treatment as usual; this will most likely be treatment for depression in parents. Additionally, they will be in regular contact with the research team, imitating the frequency of contact in the intervention group. At the end of the study, these families will also benefit from the intervention.

#### Follow-up

All families will be assessed at the same time points after being included in the study. For families in the intervention group, this translates into the following five phases: pre-intervention, post-intervention, 3 months post-intervention, 6 months post-intervention and 9 months post-intervention. Since the intervention should last around 2 months, follow-up points for families in the control group would translate into the following five stages: baseline and 2 months, 5 months, 8 months and 11 months after baseline.

### Data analysis

Data will be collected by the trained therapists and handed to the trial coordinator, who will tabulate and safely store all forms. Once participant data are coded and entered into the database, 20 % of it will be checked by a different researcher to ensure all data entry was correct.

#### Qualitative data analysis

For the interview and video data analysis, two researchers will independently code the data and key themes of the interviews will be identified. Any differences encountered by the researchers will be discussed and resolved between them. Feedback from satisfaction surveys will be used to improve the delivery and content of the intervention.

#### Quantitative data analysis

Data presentation and results will be carried out according to Consolidated Standards of Reporting Trials (CONSORT) guidelines for RCTs. An ‘intention-to-treat’ basis will be used for the analysis of all participants allocated to the study groups. Initially, a descriptive analysis of the sample will be performed. The variables will be summarized at the different time points to evaluate any changes. To evaluate the changes produced by the intervention in the different treatment groups, and on variables of a continuous nature, two-way analysis of variance will be conducted. The independent variables will be ‘time’ (pre- vs. post-treatment) and ‘group’ (intervention group vs. control group). Other exploratory analyses will be carried out to see which variables have an effect on the outcome. Logistic regression analyses will be used to adjust for predictor variables of a dichotomous outcome, such as presence or absence of psychopathology, whereas linear regression analyses will be conducted to determine the effects of predictors on the continuous variables, such as family functioning. A sensitivity analysis will be conducted to investigate the potential effects of incomplete or missing data.

### Ethics, consent and permissions

Informed consent will be obtained from all the people participating in the intervention: parents and therapists. The children will be provided with an assent form with age-appropriate language; they will be informed about the research orally and will be given the opportunity to accept or decline participation. Parental consent will also be obtained. All data will be kept safely by the principal investigator, and all data will be kept confidential. This study was submitted to and approved by the National Commission for Scientific and Technological Research ethics committee, which is an independent committee approved by the funding body, and by the North Metropolitan Health Service ethics committee, which is where the intervention will take place.

## Discussion

The recruitment process began in September 2015 with the aid of the local mental health authorities. This aim of this pilot study is to assess whether an adaptation of Beardslee’s PIP for Depression is feasible in the Chilean reality. If this pilot study were to be successful, a scale-up could be implemented across many other areas of Santiago and throughout Chile. This would be aided by that fact that many boroughs have the motivation and structure to support it.

### Strengths

This trial was met with a lot of support from the local authorities who wish to implement it as a usual intervention for children at risk for depression. Some of the strengths of this study are that the intervention will be carried out by trained psychologists and social workers who have experience working with this population. They will also be constantly supervised as an added support measure.

The fact that the intervention will be carried out at the participants’ homes is a major strength. This setting will provide the therapists with the ability to use the patient’s own environment and modify it for the family’s benefit. It will also provide the researchers with valuable qualitative information regarding whether the intervention works best in different home environments and what components of these would facilitate intervention delivery. This makes for a more personalised intervention, better fitted for each family’s needs.

Having a home-delivered intervention requires taking into account several safety challenges. Fortunately, the collaborating health centres already have the safety procedures and infrastructure set in place for home visits. Their workers therefore are fully aware of all safety procedures to be taken into account when delivering interventions in this setting.

### Difficulties

The main difficulties encountered have related to bureaucracy and time-keeping. The quality of existent electronic databases used by the health centres are also an area of concern, as many have outdated or incomplete patient information.

Recruitment is a challenge in studies of depression, and difficulties in recruiting patients into trials are well-documented [51]. It is therefore important to determine likely recruitment and retention rates for this group before embarking on a full-scale trial. This pilot study will describe the recruitment, adherence and retention rates at 11 months from baseline.

Other difficulties we will surely meet are related to family attendance. Despite the fact that interventions are carried out at the home, therapists will mostly attend during office hours, a time when at least one, if not both, parents will most likely be out working. Family structures will also be different from home to home. We expect to find many single-parent families or households in which primary caregivers are people other than the parents, such as grandparents or aunts. The intervention will have to be adjusted on the basis of each family’s needs and realities.

## Conclusions

The children of depressed parents have a higher risk of developing depression than their peers. On the one hand, treatment of these disorders, particularly pharmacological treatment, has proven controversial [52]. On the other hand, many Chileans with mental health disorders may not seek help. This is why preventive programmes that reach out to the community have such importance in many areas of Chile and the world, especially given the negative lifelong consequences and cost these disorders represent.

This pilot study is the first step towards informing the design and development of a large-scale RCT that will provide much-needed guidance on the effectiveness and efficiency of the PIP for children of parents with depression in Chile. This can, in turn, make a case for the introduction of the programme at a national level.

## Trial status

The trial has been in the recruitment stage since 15 September 2015.

## Abbreviations

BDI-II: Beck Depression Inventory-II
CBCL: Child Behaviour Checklist
CDI: Children’s Depression Inventory
DSM-IV: *Diagnostic and Statistical Manual of Mental Disorders, Fourth Edition*
E2P: Positive Parenting Scale-Second Edition
ERE: School Resilience Scale
FACES: Family Adaptability and Cohesion Evaluation Scales
IP: identified parent
M.I.N.I.: Mini International Neuropsychiatric Interview
PIP: Preventive Intervention Program
RCT: randomised controlled trial
WISC-III: Wechsler Intelligence Scale for Children-Third Edition
